# Therapeutic Potential of Retinoid X Receptor Modulators for the Treatment of the Metabolic Syndrome

**DOI:** 10.1155/2007/94156

**Published:** 2007-03-28

**Authors:** Jane A. Pinaire, Anne Reifel-Miller

**Affiliations:** Lilly Research Laboratories, Lilly Corporate Center, Indianapolis, IN 46285, USA

## Abstract

The increasing prevalence of obesity is a fundamental contributor to the growing prevalence of the metabolic syndrome. Rexinoids, a class of compounds that selectively bind and activate RXR, are being studied as a potential option for the treatment of metabolic syndrome. These compounds have glucose-lowering, insulin-sensitizing, and antiobesity effects in animal models of insulin resistance and type 2 diabetes. However, undesirable side effects such as hypertriglyceridemia and suppression of the thyroid hormone axis also occur. This review examines and compares the effects of four RXR-selective ligands: LGD1069, LG100268, AGN194204, and LG101506, a selective RXR modulator. Similar to selective modulators of other nuclear receptors such as the estrogen receptor (SERMs), LG101506 binding to RXR selectively maintains the desirable characteristic effects of rexinoids while minimizing the undesirable effects. These recent findings suggest that, with continued research efforts, RXR-specific ligands with improved pharmacological profiles may eventually be available as additional treatment options for the current epidemic of obesity, insulin resistance, type 2 diabetes, and all of the associated metabolic sequelae.

## 1. INTRODUCTION

The nuclear receptor (NR) family of transcription factors (also referred to as the steroid/thyroid hormone receptor superfamily) is quite large with approximately 150 proteins. This large superfamily may be categorized into three subgroups: *classic hormone receptors* such as the glucocorticoid, estrogen, retinoic acid, thyroid hormone, and vitamin D receptors; “*sensor*” *receptors* such as the peroxisome proliferator-activated receptors (PPARs), the liver X receptor (LXR), the farnesol X receptor (FXR), and the retinoid X receptor (RXR); and *orphan receptors* such as apolipoprotein A-I regulatory protein-1 (ARP-1) and chicken ovalbumin upstream promoter transcription factor (COUP-TF). In general, these categories describe characteristics of ligand binding to these receptors. Ligands specific for the orphan receptors, if any exist at all, have not been identified to date. Classic hormone receptors bind specific ligands with high affinity. On the other hand, a broad range of lipophilic molecules is thought to bind to the “sensor” receptors, generally with broader specificity and lower affinity as compared to hormones that bind to the classical hormone receptors. The majority of the members of this family regulates transcription of target genes by binding as homodimers or heterodimers to specific DNA sequences, called hormone response elements (HREs) or nuclear receptor responsive elements (NRREs). For the classic hormone receptors and the sensor receptors, ligand binding induces (or stabilizes) DNA binding and modulation of target gene transcription [1].

RXR is considered “promiscuous” because it forms heterodimers with several other family members that can be further classified as permissive or nonpermissive binding partners. Heterodimers formed by RXR and permissive binding partners (PPARs, LXR, and FXR) can be activated by RXR-specific ligands or by ligands specific for the binding partner. Heterodimers formed by RXR and nonpermissive partners (vitamin D and thyroid hormone receptors) can only be activated by ligands specific for the partner, but not by ligands specific for RXR [1]. However, recent evidence suggests that the concept of NR “permissivity” may require reexamination. For example, RXR has been considered a silent partner in the RXR:TR heterodimer, yet recent data indicate that this is not always true and the ability of RXR to influence the activity of the heterodimer may depend on factors such as tissue specificity, the cellular environment, or the ability of various RXR ligands to recruit coactivator or corepressor complexes to the region of the NRRE [2–4]. On the other hand, the activity of the RXR:FXR heterodimer, previously considered permissive, has recently been shown to be antagonized by ligand binding to RXR. These findings have led to the term “conditionally permissive” in describing NR heterodimers containing RXR [5].

Recent reviews have highlighted the important role of transcription and various transcription factor families, including the NRs, in the regulation of intermediary metabolism [1, 6]. Shulman and Mangelsdorf [7] recently reviewed literature demonstrating that the metabolic syndrome could be treated by altering the activity of NR heterodimers containing RXR and partners PPAR, LXR, FXR, and TR by using ligands specific for PPAR, LXR, FXR, and TR. The aim of our review, on the other hand, is to summarize available data suggesting that the metabolic syndrome could potentially be treated by altering the activity of RXR homo- and heterodimers with rexinoids, a class of compounds that bind selectively to RXR. We begin with an overview of the metabolic syndrome, its strong association with obesity and type 2 diabetes, and its prevalence. We then focus on the metabolic effects and potential therapeutic use of RXR-selective ligands. The last section discusses the utility and safety of these compounds for the treatment of the metabolic syndrome.

## 2. THE METABOLIC SYNDROME

The metabolic syndrome (also called metabolic syndrome X, syndrome X, insulin resistance syndrome, insulin resistance/hyperinsulinemia syndrome, or metabolic cardiovascular syndrome) has been extensively reviewed (see [8–11] and references therein). Briefly, insulin resistance is associated with a cluster of metabolic abnormalities that increases the risk for type 2 diabetes, cardiovascular and renal diseases, as well as some forms of cancer [8]. This cluster of metabolic abnormalities is strongly associated with obesity, predominantly visceral (abdominal) obesity, and physical inactivity and includes the following: some degree of impaired glucose homeostasis, atherogenic dyslipidemia, hypertension, an enhanced procoagulant state, and increased expression of inflammatory markers. The relationship between obesity, insulin resistance, and cardiovascular disease, recently reviewed by Reaven et al. [9] and Grundy [10], is complicated, exemplified by the fact that not all overweight or obese individuals develop insulin resistance and its associated metabolic abnormalities. However, there is little doubt that the increasing prevalence of overweight and obesity is a fundamental contributor to the rising prevalence of the metabolic syndrome (insulin resistance) and type 2 diabetes [12–14].

In a recent review, Reaven [8] differentiated the terms “insulin resistance syndrome” and “metabolic syndrome.” The insulin resistance syndrome, according to Reaven, is a term used to describe a cluster of metabolic abnormalities and related outcomes that occur more commonly in insulin-resistant/hyperinsulinemic individuals, and is not meant to identify a specific clinical entity, nor does it refer to a specific clinical diagnosis. On the other hand, the term “metabolic syndrome” is more often considered a diagnostic tool useful in the clinic for identifying individuals, presumably individuals who are insulin-resistant, who are at increased risk for cardiovascular disease and other associated outcomes. Indeed, in an effort to standardize the diagnostic criteria for the metabolic syndrome, definitions have recently been put forth by multiple national and international organizations: WHO [15, 16], ATP III [17], ACE [18], IDF [19]. The definitions are summarized in Table 1, and their utility and limitations have been compared elsewhere [20]. The clinical measures used in the definitions of the “metabolic syndrome” are typically tests that are feasible and realistic for routine clinical practice. Using the ATP III criteria, the prevalence of the metabolic syndrome has continued to increase among US adults from 28.0% as reported in the NHANES III (1988–1994) to 31.9% in the NHANES 1999-2000 [21]. Most importantly, a recent 2004 analysis revealed that the prevalence of metabolic syndrome in US children and adolescents reached 38.7% in moderately obese children and 49.7% in severely obese children [22]. These data are sobering in light of the prevalence of metabolic syndrome reported in adolescents from NHANES III (1988–1994), 6.8% among overweight adolescents and 28.7% among obese adolescents [23].

Although the definitions for the clinical diagnosis of the metabolic syndrome continue to evolve, researchers and clinicians agree on the fundamental concept of the insulin resistance/metabolic syndrome as a cluster of metabolic derangements that increase risk for type 2 diabetes, cardiovascular disease, renal disease, and other associated outcomes.

## 3. THE RETINOID-X-RECEPTOR

The NR family of transcription factors has been extensively reviewed [1]. Briefly, there are 3 RXR isotypes: RXR*α*, RXR*β*, and RXR*γ*. Each isotype is encoded by a distinct gene, and each gene is capable of generating at least 2 distinct transcripts due to alternative promoter utilization and alternative splicing. The isotypes exhibit different patterns of tissue-specific expression. RXR*α* is expressed in liver, kidney, spleen, placenta, and the epidermis; RXR*β* is expressed ubiquitously; and RXR*γ* is expressed in skeletal muscle and cardiac muscle, the anterior pituitary, and to a lesser extent in brain. Importantly, the pattern of tissue-specific expression varies widely during development. The homology of the isotypes suggests that these receptors regulate common target sequences and respond to common ligands [24, 25].

Consistent with other members of the NR family, each of the RXR isotypes has a modular domain structure [1, 24–26]. The most highly conserved region, region C, is the DNA-binding domain containing 2 zinc finger motifs, the hallmark characteristic of members of this transcription factor family. Region E, the second most conserved region, contains the ligand binding domain (LBD), the primary dimerization domain, and the ligand-dependent transcriptional activation function (AF-2). Furthermore, the LBD is the region of the receptor that binds transcriptional corepressor or coactivator complexes that mediate the effect of the receptor on transcription. The N-terminal A/B region contains a ligand-independent transcriptional activation function (AF-1). The D region is the hinge domain, allowing the DNA binding domain and the LBD to rotate. RXR homodimers and heterodimers bind to DNA targets comprised of 2 consensus hexamer motifs, or half sites, such as PuG(G/T)TCA(X), separated by a short spacer. The arrangement of these half sites and the length of the spacing between them determine the specificity of the response elements for RXR homo- or heterodimers [1, 24–26].

RXR has been shown to have diverse physiological functions using RXR knockout (KO) mouse models (reviewed in [24, 25]). Loss of RXR*α* results in much more severe phenotype than the loss of either RXR*β* or RXR*γ*. Loss of RXR*α* function in the mouse germ line results in embryonic lethality (E13.5–16.5) due to defects of the cardiac ventricles and placenta, as well as ocular abnormalities. Additional functions of RXR*α* have been identified when the receptor has been selectively deleted from specific tissues in mature animals. Loss of RXR*α* function in adipose tissue results in altered preadipocyte differentiation and resistance to obesity, though this is thought to be attributable to the absence of functional RXR*α*/PPAR*γ* heterodimers in adipose tissue. Loss of RXR*α* function in skin results in multiple phenotypic characteristics such as alopecia, hair follicle degeneration, and dermal cysts, and although some of these phenotypic characteristics are similar to the loss of function of the vitamin D receptor (VDR−/−), there are abnormalities in epidermal proliferation and differentiation in the RXR*α*−/− model that are not accounted for in the VDR−/− model. Loss of RXR*α* in the liver perturbs multiple metabolic pathways mediated by LXR*α*, PPAR*α*, CAR*β*, PXR, and FXR. Interestingly, many, but not all, of the defects in lipid metabolism in liver-specific RXR*α*−/− mice are similar to those in LXR−/− mice, suggesting that RXR*α*:LXR plays an important role in hepatic lipid metabolism. The phenotype of RXR*α*−/− in liver also shows similarities to PPAR*α*−/− mice, suggesting that the phenotype in each is attributable to a loss of functional RXR*α*:PPAR*α* heterodimers. Furthermore, when RXR*α* is absent in the liver of adult mice, the regenerative capacity of hepatocytes is impaired, and the hepatocytes have a shorter lifespan compared to wild-type animals. Absence of RXR*α* in prostate produces a marked alteration in the profile of secretory proteins as well as preneoplastic lesions (reviewed in [24, 25]).

Loss of either RXR*β* or RXR*γ* in the mouse germ line is not embryonic lethal. Approximately 50% of RXR*β*−/− mice die before or at birth, but the animals that do survive appear to be normal except that the males are sterile. RXR*γ*−/− mice develop normally and appear similar to wild-type animals, except they have higher serum T4 and TSH levels and higher metabolic rates compared to wild-type animals (reviewed in [24, 25]).

Because RXR has been shown to play a role in diverse physiological processes including cell proliferation, differentiation, and apoptosis and metabolism, RXR isotypes have been referred to as “master regulators” [24, 25]. Therefore, compounds that alter the activity of RXR also have the potential to alter multiple physiological and metabolic pathways, with the potential of both beneficial and deleterious effects. Indeed, in animal models of insulin resistance and diabetes, rexinoids have been shown to have beneficial glucose-lowering, insulin-sensitizing, and antiobesity effects, while at the same time raising triglyceride (TG) levels and suppressing the thyroid hormone axis, side effects that have limited the development of these compounds as therapeutic agents for the treatment of type 2 diabetes and insulin resistance. In the following sections we review the metabolic effects of four different rexinoids, one of which is a selective RXR modulator.

## 4. EFFICACY OF REXINOIDS AS THERAPEUTIC AGENTS

### 4.1. LGD1069 (Bexarotene; Targretin, Ligand Pharmaceuticals Inc., Calif, USA)

LGD1069 was the first compound found to be a potent and highly specific ligand for RXR [27]. LGD1069 has poor binding affinity for RAR isoforms *α*, *β*, or *γ* (Kd >1000 nM for all isoforms). On the other hand, LGD1069 binds with high affinity to RXR *α*, *β*, and *γ*: Kd values are 14 ± 3, 21 ± 4, and 29 ± 7 nM, respectively, see [28]. LGD1069 is used clinically for the treatment of cutaneous T-cell lymphoma, though its use for treating other cancers is currently being investigated [29]. LGD1069 has been shown to lower glucose and insulin levels in the ob/ob mouse to degrees similar to rosiglitazone (ROSI) [30]. At 53 days of age, the average fasting glucose and insulin concentrations in ob/ob mice are 262 mg/dL and 12–18 ng/ml, respectively. Over the course of 2 weeks, this hyperglycemia and hyperinsulinemia continues to worsen. Treatment of 53-day old female ob/ob mice with LGD1069 resulted in a 45% reduction of fasting plasma glucose (55% reduction with ROSI) and a 30% reduction of fasting plasma insulin (15% reduction with ROSI) [30]. Most of the published data regarding LGD1069 describes effects on lipids and TG, and that data is reviewed in following sections.

### 4.2. LG100268

As a selective ligand for RXR, LG100268 (LG268) has poor binding affinity for RAR isoforms *α*, *β*, or *γ* (Ki >1000 nM for all isoforms). On the other hand, LG268 binds with high affinity to RXR *α*, *β*, and *γ*: Ki values are 3.2, 6.2, and 9.7 nM, respectively, see [31].

#### 4.2.1. Effects on glucose and insulin resistance

LG268 has also been shown to lower glucose and insulin levels in the ob/ob mouse. Treatment of 53-day old female ob/ob mice with LG268 for 2 weeks resulted in a 48% reduction of fasting plasma glucose (55% reduction with ROSI) and a 59% reduction of fasting plasma insulin (15% reduction with ROSI) [30]. A glucose/insulin tolerance test showed significant reductions of both the area under the glucose curve and the area under the insulin curve with LG268, and insulin resistance was reduced approximately 75% [30].

By the age of 16 weeks, the C57BL/KsJ strain of db/db mice demonstrates a number of characteristics of progressed type 2 diabetes and pancreatic *β*-cell dysfunction; compared to 6-week old animals, serum insulin levels are nearly 10-fold lower and serum glucose levels and glycohemoglobin are nearly doubled. Serum glucose, glycohemoglobin, and fibrinogen, commonly elevated in insulin resistance, were reduced similarly by ROSI and LG268, and insulin content in pancreatic islets increased significantly with both compounds. The effects of ROSI and LG268 on body and organ weights were compared. Between weeks 14 and 16, mean body weight change in vehicle-, ROSI-, and LG268-treated animals was −2.7, +3.2, and +0.5 grams, respectively. Unlike ROSI, LG268 did not induce adipose tissue hypertrophy. Furthermore, LG268 produced several effects on the liver not observed with ROSI: hepatomegaly, increased peroxisome number, fatty infiltration, and induced expression of microsomal lauric acid hydroxylase. Therefore, compared with ROSI, LG268 had very similar effects on glucose-lowering, pancreatic insulin content, and serum fibrinogen levels, but distinctly different effects on body weight and liver, effects likely mediated via the RXR:PPAR*α* pathway [32].

To further distinguish the mechanism of rexinoids as glucose-lowering and insulin sensitizing compounds from that of TZDs, Ahuja et al. examined the effect of LG268 on mRNA levels of three TZD-inducible genes shown to contain a PPRE for the RXR:PPAR*γ* heterodimer: mitochondrial carnitine palmitoyl transferase (MCPT), steroyl coenzyme A desaturase 1 (SCD1), and fatty acid translocase (FAT). Message levels were compared in adipose tissue, skeletal muscle, and liver. To summarize, both compounds induced mRNA of all three genes: ROSI induced message levels in adipose tissue and LG268 induced the mRNAs in liver. The compounds had similar actions in skeletal muscle. Similar to what was reported by Lenhard et al. [32], ROSI and LG268 have similar effects on glucose-lowering, though their mechanisms may be quite distinct. The authors concluded that rexinoids do not function as simple “TZD mimetics” in vivo, as previously suggested, and though the RXR:PPAR heterodimer has been shown to be permissive using in vitro transfection experiments with synthetic reporter constructs, rexinoids have a pharmacological profile distinct from TZDs [33].

To further investigate how rexinoids and TZDs differ in mediating their glucose-lowering and insulin-sensitizing effects, Shen et al. compared the effects of ROSI and LG268 on insulin signaling in muscle of db/db mice [34]. As reported by the other studies, ROSI and LG268 had similar effects on glucose-lowering. However, ROSI and LG268 were demonstrated to have distinctly different effects on components of the insulin signaling pathway in muscle. In quadricep, neither compound had any effect on insulin receptor mRNA or protein levels, or on insulin receptor tyrosine phosphorylation. However, while ROSI increased expression (mRNA and protein) of c-Cbl-associated protein (CAP), LG268 induced insulin-stimulated tyrosine phosphorylation of the insulin receptor substrate-1 (IRS-1). In extensor digitorum longus (EDL) muscle, ROSI induced both basal and insulin-stimulated tyrosine phosphorylation of C-Cbl, while LG268 induced insulin-stimulated Akt phosphorylation. In addition, LG268 suppressed IRS-1 Ser307 phosphorylation, which has been implicated in insulin resistance. Taken together, these data provided clear evidence that TZDs and rexinoids exert their effects through different mechanisms. In muscle, TZDs mediated their effects through the CAP/c-Cbl pathway, and rexinoids exerted their effects through the IRS-1/Akt pathway, suggesting that LG268 does not mediate its effects in muscle via binding to the RXR:PPAR*γ* heterodimer. Although LXR ligands have effects similar to rexinoids, the authors point out that this too is unlikely because LXR ligands had no effect on Akt phosphorylation in these animals. Therefore, the authors speculate that LG268 may mediate its effects in muscle through PPAR*α* or PPAR*δ*, or possibly through the RXR:RXR homodimer [34].

#### 4.2.2. Effects on obesity

Three studies using the Zucker fatty rat model have demonstrated antiobesity effects of rexinoids [35–37]. Emilsson et al. compared the effects of ROSI and LG268 on food consumption, body weight, and the expression of uncoupling protein (UCP) isoforms UCP-1, UCP-2, and UCP-3 [37]. ROSI significantly increased food consumption, but had no significant effect on body weight change. In contrast, LG268 significantly decreased food consumption and body weight. UCP-1 mRNA in brown adipose tissue (BAT) was significantly induced by LG268, but not by ROSI. Neither ROSI nor LG268 had any effect on UCP-2 mRNA in BAT, white adipose tissue (WAT), muscle, or brain. UCP-3 mRNA was induced by ROSI and LG268 in BAT and WAT, but in muscle only ROSI induced UCP-3. Thus, in the Zucker fatty rat, rexinoids have antiobesity effects, whereas TZDs do not. Furthermore, the authors speculated that LG268 may promote thermogenesis due to the upregulation of UCP-1 in BAT [37].

To further investigate the mechanisms underlying the antiobesity effects of rexinoids, Ogilvie et al. [36] evaluated body weight and cumulative food consumption over 42 days in female Zucker fatty rats treated with ROSI or LG268. LG268 significantly reduced body weight and cumulative food consumption whereas ROSI increased both body weight and consumed food. Body composition analysis revealed that ROSI increased fat mass, but LG268 reduced fat mass in both fatty and lean animals. Neither compound had an effect on lean body mass. The effects of ROSI and LG268 on adipogenesis and apoptosis in subcutaneous and mesenteric (ovarian) fat were compared. In ovarian fat, there was no difference between ROSI and LG268 in their effects on apoptosis and adipogenesis. In contrast, in subcutaneous fat, LG268 more strongly induced apoptosis and was a weaker inducer of adipogenesis compared with ROSI, suggesting that the LG268-mediated reduction of fat mass was at least in part due to increased apoptosis in subcutaneous adipose tissue. To determine whether decreased food consumption and body weight observed with LG268 treatment was due to adverse toxicological effects, dynamic feeding behavior before, during, and after treatment with LG268 was examined. The authors hypothesized that if LG268 had adverse toxicological effects, the animals would forgo meals (decreased meal frequency); on the other hand, if LG268 truly had antiobesity properties, the animals would become satiated after consuming less food (decreased food consumption). Indeed, LG268 was associated with decreased meal size. Finally, because RXR is present in hypothalamic satiety centers, the authors tested whether LG268 acts centrally in the brain. Injection of LG268 directly into the cerebral ventricles produced reductions of cumulative body weight gain and daily food consumption similar to that observed with oral administration of LG268, suggesting that LG268 mediated its antiobesty effects through the central nervous system. Most surprising was the observation that the TG-raising effect of LG268 (discussed in greater detail below) was abolished with ICV administration. These observations led the authors to conclude that LG268 not only regulates feeding behavior and body weight in a manner completely distinct from TZDs, through the central nervous system, but that the antiobesity action of rexinoids may be separable from its effects on TG [36].

### 4.3. AGN194204

As a selective ligand for RXR, AGN194204 has poor binding affinity for RAR isoforms *α*, *β*, or *γ* (Kd >30 K nM for all isoforms). On the other hand, AGN194204 binds with high affinity to RXR *α*, *β*, and *γ*: Kd values are 0.4, 3.6, and 3.8 nM, respectively, see [38].

#### 4.3.1. Effects on glucose and insulin resistance

Li et al. [39] examined the metabolic effects of AGN194204 in female Zucker diabetic fatty rats (ZFF rats). After being fed a high-fat diet (48% fat and 16% protein) for 3-4 weeks, these animals were treated with troglitazone (TROG) or AGN194204. AGN194204 and TROG had very similar effects on lowering serum glucose and insulin. A hyperinsulinemic-euglycemic clamp indicated that the insulin-sensitizing effects of AGN194204 occurred predominantly in the liver, while TROG exerted its insulin-sensitizing effect in the liver and in peripheral tissues. The effects of AGN194204 and TROG on components of the insulin signaling pathway in liver and skeletal muscle were compared. In liver, AGN194204 increased IRS-2 protein levels before and after treatment with insulin, and increased insulin-stimulated Akt phosphorylation following insulin treatment. In skeletal muscle, TROG stimulated Akt phosphorylation and produced a small but consistent increase in IRS-1 protein before and after insulin treatment. These results are consistent with liver being the primary target of insulin-sensitizing effect of AGN194204, and with skeletal muscle being an important target of the insulin-sensitizing effects of TROG.

### 4.4. LG101506

LG101506 (LG1506) has poor binding affinity for RAR isoforms *α*, *β*, or *γ*: Ki values are 2746 ± 395, 3516 ± 420, and >10 000 nM, respectively. On the other hand, LG1506 binds with high affinity to RXR *α*, *β*, and *γ*: Ki values are 3.0 ± 0.8, 9.0 ± 1.7, and 11.0 ± 3.6, respectively, see [40].

#### 4.4.1. Effects on glucose and insulin resistance

Unlike other RXR-selective ligands, LG1506 binding to the RXR receptor induces a conformation that results in selective activation of RXR:PPAR*γ*, RXR:PPAR*α*, and RXR:PPAR*δ*, but not RXR:RAR, RXR:LXR, or RXR:FXR heterodimers [40]. The glucose-lowering and insulin-sensitizing effects of LG1506 were comparable to those of LG268 and ROSI in the female Zucker fa/fa rat. However, when administered as a single agent, LG1506 had no significant effect on body weight, while, as reported previously, body weight was increased with ROSI and decreased by LG268. When coadministered, however, LG1506 completely blocked the weight gain observed with ROSI.

### 4.5. Summary of the effects of rexinoids on glucose, insulin resistance, and obesity

The effects of RXR-specific ligands on glucose, insulin resistance, and obesity are summarized in Table 2. Review of the literature describing the antidiabetic effects of rexinoids shows that RXR-selective ligands do not mediate their glucose-lowering and insulin-sensitizing effects by merely “mimicking” the effects of TZDs. Initially thought to synergize the actions of TZDs through binding to the RXR component of the RXR:PPAR*γ* heterodimer, recent evidence clearly demonstrates that these two classes of compounds mediate their actions via distinct mechanisms. Both Shen et al. [34] and Li et al. [39] have demonstrated that rexinoids and TZDs act through distinct pathways of insulin signaling. In addition, while both rexinoids and TZDs remodel adipose tissue, rexinoids have antiobesity properties whereas TZDs have the opposite effect. Futhermore, Ogilvie et al. [36] have demonstrated that the rexinoid LG268 mediates its antiobesity effects through the central nervous system. While the rexinoid LG1506 was shown to be weight-neutral when administered as a single agent in the Zucker fa/fa rat model [40], it completely blocked weight gain when coadministered with ROSI. Therefore, compared to TZDs, glucose-lowering, insulin-sensitizing agents currently used for the treatment of insulin resistance and type 2 diabetes, the rexinoids, with further development, may eventually offer the advantage of promoting weight loss, though being weight-neutral would be an advantage over TZDs. Furthermore, if rexinoids were to be used in combination with TZDs, this may offer even greater efficacy and limit the weight gain characteristic of TZDs.

## 5. SAFETY OF REXINOIDS AS THERAPEUTIC AGENTS

### 5.1. Elevation of triglyceride levels

Plasma triglyceride levels are maintained at normal levels in fasted animals due to the established equilibrium between the rate of hepatic secretion of very low density lipoproteins (VLDL) versus the rate of VLDL clearance. VLDL particles are cleared by lipoprotein lipase (LPL) activity present in the tissue vascular beds and hepatic lipase present in the liver [43].

Hypertriglyceridemia was initially seen in patients receiving retinoic acid isomers that are known to activate both RARs and RXRs. Administration of RAR-specific retinoids results in hypertriglyceridemia in rodents [44] and in humans [45]. Similarly, patients in a phase I clinical trial for advanced cancer treated with the RXR-selective agonist LG1069 experienced elevated triglyceride levels [46, 47].

### 5.2. LGD1069

Recent studies have investigated the physiological and molecular bases of the hypertriglyceridemia associated with LGD1069 treatment [41, 48, 49]. Treatment of ZDF rats with a broad range of LGD1069 doses (0.3, 1, 3, 10, or 30 mg/kg) for 14 days produced a nearly linear dose-dependent increase in serum TG levels, with no change in total cholesterol levels. Lipoprotein profile analysis revealed no significant change in the IDL or LDL fractions with LGD1069 treatment, and no significant change in the HDL fraction, though HDL particles were less heterogeneous and more bulky relative to control. However, LG1069 significantly increased the VLDL fraction. The authors investigated whether LG1069 reduced VLDL clearance by affecting lipoprotein lipase (LPL). LPL activity was potently suppressed by LG1069 in heart and skeletal muscle, with skeletal muscle being the most sensitive. Importantly, this suppression of LPL activity in skeletal muscle and cardiac tissue did not correspond to reduced LPL mRNA levels, as LG1069 had no effects on LPL mRNA levels in either tissue. On the other hand, LG1069 had no effect on LPL mRNA or activity in adipose tissue. The authors proposed that the hypertriglyceridemia observed in LG1069-treated animals was due to elevated VLDL caused by a primary defect in LPL-dependent catabolism. Furthermore, the authors speculated that skeletal muscle in particular may be an important rexinoid target tissue [41].

In contrast, when Ouamrane et al. [48] treated male C57BL/6J mice with LGD1069 or fenofibrate, no effect of LGD1069 on serum TG levels was observed. As expected, fenofibrate decreased serum TG approximately 50%, and no change in serum cholesterol was observed with either treatment. When this experiment was repeated using age-matched male PPAR*α*-deficient mice (C57BL/6J background), LG1069 increased serum TG levels approximately 3-fold and the TG-lowering effect of fenofibrate was abolished. To further examine the involvement of PPAR*α* in the effects of LGD1069, the ability of LGD1069 to induced hepatomegaly was examined, as was the ability of LGD1069 to induce mRNA of genes known to be responsive to peroxisome proliferators (PPs): CYP4A (cytochrome P450 4A) and PDK4 (pyruvate dehydrogenase kinase 4). In wild-type animals, LG1069 and fenofibrate induced CYP4A mRNA and hepatomegaly. In PPAR*α*-deficient animals, the ability of fenfibrate to induce hepatomegaly was lost while the ability of LG1069 to induce hepatomegaly remained intact, though both fenofibrate and LG1069 failed to induce CYP4A in liver. In wild-type animals, LG1069 induced PDK4 mRNA in both heart and kidney, whereas LGD1069 induced PDK4 mRNA only in the heart tissue of PPAR*α*-deficient animals. These observations led the authors to propose that rexinoids such as LGD1069 mediate their physiological effects through both PPAR*α*-dependent pathways (induction of CYP4A mRNA in liver and PDK4 mRNA in kidney) and PPAR*α*-independent pathways (hepatomegaly and induction of PDK4 mRNA in cardiac tissue). Still, the exact mechanism explaining why LGD1069 increased serum TG by 3-fold in the PPAR*α*-deficient animals and not at all in the wild-type animals remained unclear. The authors speculated that two separate pathways may account for the effects of LGD1069 on serum TG in this animal model: a PPAR*α*-independent pathway may account for the TG-raising effect of LGD1069, while a PPAR*α*-dependent pathway would decrease serum TG via activation of the RXR:PPAR*α* heterodimer. Thus, TG levels in the wild-type animal would reflect the relative activity of both pathways [48].

Although LGD1069 elevates serum TG in ZDF rats, very recent data demonstrate that LGD1069 inhibits atherosclerosis progression in the apolipoprotein E2 knockin (Apo E2-KI) mouse model [49]. Compared with the Apo E knockout (KO) model, which is characterized by isolated hypercholesterolemia, the Apo E2-KI mouse model develops a mixed dyslipidemia (elevated TG and hypercholesterolemia) more commonly found in humans. Female Apo E2-KI mice (C57BL6 background) were fed a Western-style diet (0.2% cholesterol and 21% fat) supplemented with or without LG1069 (0.018% wt/wt) for 11 weeks. Oil-Red-O staining of atherosclerotic lesions in the aorta demonstrated that LGD1069 treatment significantly reduced lesion area, though LGD1069 increased plasma TG concentrations over 50%. TG was associated with the VLDL fraction and LGD1069 produced a significant reduction of plasma total cholesterol that correspondeds to a reduction in non-HDL-C (IDL and LDL cholesterol). LGD1069 significantly decreased plasma Apo B, though no change in liver Apo B mRNA was observed. Furthermore, LGD1069 significantly induced LDL receptor mRNA in liver (approximately 2-fold). LGD1069 was also shown to significantly decrease intestinal cholesterol absorption, as evidenced by reduced mRNA levels of Niemann-Pick C1-Like1 (NPC1L1) and CD13, genes recently identified as critical components of the intestinal cholesterol absorption machinery, and ABCA1 in both duodenum and jejunum. LGD1069 also significantly increased ABCA1 and ABCG1 mRNA levels in the aortic sinus. Peritoneal macrophages obtained from control Apo E2-KI mice showed significant lipid accumulation; however, LGD1069 treatment prevented lipid accumulation in vivo and significantly enhanced Apo AI- and HDL-mediated cholesterol efflux from these macrophages in vitro. Although the mechanism(s) through which LGD1069 exerts these effects remains unclear, the authors suggest that the RXR:LXR pathway may play an important role, but emphasize that LGD1069 may selectively operate through RXR:LXR in a tissue-specific and gene-specific manners. In contrast to the ZDF rat model [41], these results suggest that LGD1069-mediated hypertriglyceridemia in the Apo E2-KI mouse model is countered by a decrease in non-HDL cholesterol (IDL and HDL), a corresponding increase in hepatic LDL receptor mRNA, decreased intestinal cholesterol absorption, and increased Apo A- and HDL-dependent cholesterol efflux [49].

### 5.3. LG100268

Studies of the effects of LG268 on TG are difficult to interpret due to conflicting results. Studies using db/db mice showed that LG268 lowers TG [30, 32, 34, 50] while a study using ZDF rats showed that LG268 increases TG [41]. Mukherjee demonstrated that gemfibrozil and LG268, as single agents, significantly reduced plasma TG, while coadministration produced an additive reduction. Similarly, genfibrozil and LG268 each increased plasma HDL-C levels, while coadministration produced an additive increase. The authors concluded that the RXR:PPAR*α* heterodimer was the common target of genfibrozil and LG268 in TG-lowering and HDL-raising [50]. In a separate study, Mukherjee et al. [30] suggested that the RXR:PPAR*γ* heterodimer was the common target of ROSI and LG269 in TG-lowering. ROSI and LG268, as single agents, significantly reduced serum TG, though coadministration produced an additive decrease [30]. In contrast, Lenhard et al. reported that although ROSI and LG268 displayed similar effects on reducing serum glucose and glycohemoglobin in db/db mice with progressed pancreatic *β*-cell dysfunction, LG268 lowered TG, but not as potently as ROSI [32]. This suggested that the TG-lowering effects of LG268 in these animals were not mediated by the RXR:PPAR*γ* heterodimer [32]. To further investigate these discrepant results, Davies et al. [41] treated ZDF rats with either ROSI or LG100268 for 14 days. ROSI maximally decreased serum TG levels at day 3, while LG268 steadily increased TG levels throughout the treatment period. When a single dose of LG268 was administered to nonobese, nondiabetic Sprague Dawley rats, after a lag period of 60 minutes, serum TG rose rapidly and remained elevated for approximately 6 hours before returning to normal by 24 hours. The LG268-mediated TG increase was abolished by pretreatment with actinomycin D. Post-heparin plasma LPL activity was significantly decreased 3 hours after administration of the single LG268 dose. To further characterize the effect of LG268 on LPL activity, C2C12 differentiated mouse myocytes were stably transfected with an LPL expression vector. In vitro LPL activity was potently suppressed by LG268 and this suppression was abolished by cotreatment with actinomycin D. Furthermore, in vitro LPL activity was not suppressed by WY14,643 or ROSI, suggesting that LG268 mediates these effects via pathways distinct from PPAR*α* and PPAR*γ* [41].

### 5.4. AGN194204

The rexinoid AGN194204 significantly increased serum TG concentrations while TROG decreased TG in female Zucker diabetic fatty rats (ZFF) [39]. A time course examining the effect of AGN194204 on serum TG demonstrated that TG levels are induced by AGN194204 approximately 3-fold after only 1 day of treatment and remain elevated at that level until day 3. By day 7, serum TG levels have begun to decrease to levels less than 2-fold control. Liver TG content increases less than 2-fold with AGN194204, and those levels remain stable throughout a 7-day treatment period. Affymetrix gene chips were utilized to gain further insight into the metabolic actions of AGN194204. Mice were treated with vehicle or AGN194204 and total liver RNA hybridized to the chip. As described in detail in [39], the data were analyzed using a web-based expression analysis program. To summarize, two gene expression networks were found to be significantly altered in response to treatment with AGN194204. One network centered on the increased expression of SREBP-1c and genes such as FAS, ACO, 3-keto-CoA thiolase, and FABP; the second network consisted of genes containing G-protein subunits coupled to the glucagon receptor as well as to FoxA. Glucagon receptor mRNA levels in liver did not change in response to either AGN194204 or TROG treatment, however, FoxA2 and FoxA3 mRNA levels were significantly reduced in liver following treatment with AGN194204. Based on these findings, the authors speculated that at least one mechanism explaining AGN194204-mediated hypertriglyceridemia (in addition to the possibility of AGN194204 activating the RXR:LXR heterodimer) could be the AGN194204-mediated increase of IRS-2 and decrease of FoxA2 in liver. FoxA2 increases the expression of fatty acid oxidizing enzymes in the liver, and insulin inhibits FA oxidation in part by sequestering FoxA2 in the cytoplasm. Therefore, AGN194204 treatment would ultimately inhibit fatty acid oxidation and increase SREBP-1c and other enzymes involved in de novo TG synthesis.

### 5.5. LG101506

Compared with LG268, which potently induced TG in nonobese, nondiabetic Sprague Dawley rats, LG1506 had no effect on TG levels [40]. TG levels were significantly elevated 2 hours after administration of a single dose of LG268, whereas TG levels were similar to control 2 hours after treatment with LG1506. In Zucker fa/fa rats, similar results were observed in that levels that were potently induced by LG268 over a 14-day treatment period, and TG levels were increased, though not significantly, by treatment with LG1506. Interestingly, the greatest effect of LG1506 on TG levels was observed at a lower dose of 3 mg/kg, and this effect was most evident on day 7. This curious observation was further investigated in lean and obese Zucker rats treated with 1, 3, or 30 mg/kg LG1506 for 7 days. Indeed, low doses (1 and 3 mg/kg) of LG1506 induced TG by day 3 in both lean and obese animals with maximal elevations at day 7, while TG levels after treatment with higher doses of LG1506 (30 mg/kg) remained similar to control levels for the duration of the treatment period. These findings led the authors to speculate that LG1506 may affect multiple pathways that regulate TG metabolism: pathways that increase TG levels at low doses of LG1506 and pathways that oppose the TG-raising effects at higher doses of LG1506. The authors add that the complexity of the effects of LG1506 on TG requires further investigation before agents such as LG1506 can be used for the treatment of insulin resistance and type 2 diabetes, however, progress has been made in developing RXR-selective agonists that maintain glucose-lowering properties with the potential to minimize unwanted side effects.

### 5.6. Suppression of the thyroid hormone axis

Regulation of thyroid hormone levels is the result of a complex interaction involving the hypothalamic-pituitary-thyroid axis. Thyroid-stimulating hormone (TSH), also known as thyrotropin, is a glycoprotein hormone that stimulates development of the thyroid gland and also its secretory activity. Thyrotropin-releasing hormone (TRH) is produced by the hypothalamus and triggers TSH release from the anterior pituitary. TSH release is inhibited by negative feedback exerted by rising blood levels of thyroid hormone acting on both the pituitary and the hypothalamus. Thyroid hormone is actually two active iodine-containing hormones, thyroxine (T4) and triiodothyronine (T3). Thyroxine is the major hormone secreted by the thyroid gland, with the majority of T3 formed at the target tissue by conversion of T4 to T3 [55].

The relationship between alterations in thyroid hormone levels and retinoid administration has been known for many years. In 1947, Simkins described the use of high doses of vitamin A (retinol) for the treatment of hyperthyroidism [56]. Through extensive conversion in vivo, retinol is modified to retinaldehyde, all-trans retinoic acid, and finally 13-cis and 9-cis retinoic acid, which are known to activate genes through the RAR and RXR pathways. Many years later, central hypothyroidism with significant suppression of serum TSH levels was noted in patients with refractory or persistent early-stage cutaneous T-cell lymphoma following treatment with the synthetic RXR-selective retinoid LG1069 [51, 52].

The mechanism for RXR-induced thyroid hormone alterations was investigated in preclinical studies using the RXR-selective ligands, LG268 and AGN194204. After a single administration of LG268 to Sprague Dawley rats, a rapid and statistically significant decrease in TSH levels was seen acutely, 0.5 to 1 hour after treatment. In contrast, total T3 and T4 levels declined more gradually reaching statistical significance 24 hours after compound administration. Further studies investigating the mechanism for TSH suppression showed that neither TSHb mRNA nor TSH protein levels were altered; however, LG100268 treatment reduced TRH-stimulated TSH secretion by 54% [53]. Similar findings were seen with another high affinity RXR-selective ligand, AGN194204. When administrated to female Zucker rats and nondiabetic littermates, AGN194204 decreased TSH levels by 70–80%, which was followed by a decrease in T3 and T4 levels. In diabetic mice, AGN194204 caused a time-dependent decrease in TSH levels after one day of treatment proceeding the fall in T4 levels that was significant at three days after the initiation of treatment [42]. More recent studies with LG268 in mice and using a thyrotrope-derived cell line showed that rexinoids directly suppress TSH secretion, TSH*β* mRNA levels, and promoter activity, but no direct effect on hypothalamic TRH levels was seen. These studies also demonstrated that any of the RXR isotypes (*α*, *β*, or *γ*) can mediate TSH suppression by rexinoids, but the RXR*γ* isotype is most efficient at mediating this response [54].

### 5.7. Summary of the effects of rexinoids on hypertriglyceridemia and suppression of the thyroid hormone axis

The effects of RXR-specific ligands on hypertriglyceridemia and suppression of the thyroid hormone axis are summarized in Table 3. By reviewing the literature investigating the effects of rexinoids on lipids, particularly hypertriglyceridemia, it is clear that these compounds have diverse and complex effects; furthermore, the ability of rexinoids to lower or raise TG levels may depend on a number of factors, including species and strain of the animal model used and the characteristics of the individual ligand. For example, LG1069 increased TG in the ZDF rat [41], had no effect on TG levels in the C57BL/6J mouse [48], and increased TG levels significantly in the apoE2-KI mouse model [49]. Interestingly, using the apoE2-KI model, Lalloyerc et al. demonstrated that LG1609 significantly inhibited the progression of atherosclerosis despite TG levels being increased over 50% [49]. In addition to LG1069 significantly reducing atherosclerotic lesion area in the apoE2-KI model, the rexinoid also resulted in a number of additional beneficial effects on lipid metabolism: significant reduction of non-HDL cholesterol, reduction of plasma apoB levels, induction of hepatic LDL receptors, reduced intestinal cholesterol absorption, and enhanced apoAI- and HDL-mediated cholesterol efflux. These findings prompted a rather controversial speculation by the authors: perhaps elevated TG, long thought to be an independent risk factor for cardiovascular disease, may not increase the risk for cardiovascular disease when associated with a concomitant decrease in non-HDL cholesterol. LG268 was shown to consistently lower TG in the db/db mice model and increase TG in ZDF rat [30, 32, 34, 41, 50]. Li et al. showed that AGN194204 increased TG levels in ZFF rats [39], and speculated on the mechanism of AGN194204-mediated hypertriglyceridemia by using data obtained from Affymetrix gene chip analysis. Finally, Leibowitz et al. provided data on LG1506, a selective RXR modulator that preferentially activates RXR heterodimers with PPAR*α*, PPAR*γ*, and PPAR*δ* [40]. LG1506 was shown to increase TG at low doses while having no effect on TG levels at higher doses that are efficacious for the antidiabetic effects of LG1506.

Suppression of the thyroid hormone axis has been observed in patients receiving LG1609 for the treatment of cutaneous T-cell lymphoma [51, 52]. The mechanism of LG268-mediated hypothyroidism was investigated in the rat [53] and mouse [54]. Sharma et al. [54] report that LG268 exerts multiple effects on the hypothalamic-pituitary-thyroid axis, and Ogilvie et al. [36] demonstrated that hypertriglyceridemia and suppression of the HPT axis are separable from effects on body weight and food intake. Whether administered orally or ICV, LG268 reduced food intake and body weight; on the other hand, LG268 only increased TG levels and suppressed total T4 levels when administered orally. Macchia et al. [42] demonstrated that AGN194204 caused central hypothyroidism independently of TR, the main mediator of hormone-induced TSH suppression. Finally, Leibowitz et al. [40] showed that suppression of the HPT axis could be minimized with the selective RXR modulator, LG1506.

## 6. FUTURE DIRECTIONS

The number of individuals with obesity and type 2 diabetes is growing at epidemic rates, reaching younger populations and expanding into newly emerging industrialized nations. RXR-specific ligands have potent glucose-lowering, insulin-sensitizing, and antiobesity effects in animal models of obesity, insulin resistance, and type 2 diabetes. As the mechanisms underlying the adverse side effects of the RXR agonists become better understood, the potential to enhance the beneficial effects and minimize (or even abolish) the negative side effects of RXR ligands may be feasible. By eliminating the alterations in the thyroid hormone axis and modifying the triglyceride liabilities, the selective RXR modulator approach in the example of LG101506 is promising. More extensive studies are clearly needed, but the global epidemic of obesity and type 2 diabetes highlights the opportunity to further explore the therapeutic potential of retinoid X receptor modulators for the treatment of the metabolic syndrome.

## Figures and Tables

**Table 1:** Comparison of definitions of the metabolic syndrome.

Metabolic parameter	WHO [15, 16]	ATP III [17]	ACE‡ [18]	IDF [19]

Elevated TG (mg/dL)	≥ 150	≥ 150	≥ 150	≥ 150 or treatment for elevated TG

Low HDL-C* (mg/dL)	< 39 (female)	< 50 (female)	< 50 (female)	< 50 (female) < 40 (male) or treatment for low HDL-C
	< 35 (male)	< 40 (male)	< 40 (male)	

Elevated blood pressure (mm Hg)	≥ 140/90	≥ 130/85	≥ 130/85	≥ 130/85 or treatment for previously diagnosed HTN

Elevated fasting glucose (mg/dL)	—	≥ 110	110–125	≥ 100 or previously diagnosed diabetes

Elevated 2-hour post-challenge glucose (mg/dL)	—	—	> 140	—

Waist circumference* (cm)	—	> 88 (female)	—	—
		> 102 (male)		

Waist-to-hip ratio*	> 0.85 (female)	—	—	—
	> 0.90 (male)			

High BMI	> 30 kg/m2	—	Obesity is included in a list of factors that increase the likelihood of insulin resistance **	—

Microalbuminuria	≥ 20 *μ*g/min or albumin-to-creatinine ratio ≥ 30 mg/g	—	—	—

Definition	Diabetes, impaired fasting glucose, impaired glucose tolerance, or insulin resistance plus 2 or more of the above	Three or more of the above	Risk factors** plus two or more of the above	Central obesity (ethnic-specific cut points) ≥ 94 cm (female) ≥ 80 cm (male) plus two or more of the above

Table modified from [12, 21]. Abbreviations: WHO, World Health Organization; ATP III, National Cholesterol Education Program Adult Treatment Panel III; ACE, American College of Endocrinology; IDF, International Diabetes Federation; HTN, hypertension.

‡ ACE uses term “insulin resistance syndrome”

* Gender-specific parameters

** Risk factors include overweight (BMI > 25 kg/m2 or waist circumference > 40 inches for men and > 35 inches for women), sedentary lifestyle, age > 40 years, non-Caucasian, family history of type 2 diabetes, hypertension, or cardiovascular disease, and personal history of polycystic ovarian syndrome, gestational diabetes, acanthosis nigricans, or nonalcoholic steatohepatitis.

**Table 2:** Summary of the effects of RXR-specific ligands on glucose, insulin resistance, and obesity.

RXR ligand	Effects on glucose [references]	Effects on insulin or insulin resistance [references]	Effects on body weight [references]	Effects on insulin signalling [references]

LGD1069	↓ [30]	↓ [30]	No change [30]	

LG100268	↓ [30, 32–34, 40, 41]	No change [32]	No change [30, 32, 34]	IRS-1/Akt pathway and decreased
		↓ [30, 33–36, 40]	↓ [35–37, 40]	IRS-1 Ser307 phosphorylation in muscle [34]

AGN194204	↓ [39]	↓ [39]	No change [42]; ↑ [39]	↑ IRS-2 protein expression before and after insulin treatment; increase in insulin-stimulated Akt phophorylation in liver [39]

LG101506	↓ [40]	↓ [40]	No change [40]	

**Table 3:** Summary of the effects of RXR-specific ligands on hypertriglyceridemia and suppression of the thyroid hormone axis.

RXR ligand	Effects on triglycerides [references]	Effects on thyroid hormone axis [references]

LGD1069	No change in PPAR*α* WT mice;	Human data: ↓ ([47, 51, 52]; reviewed in [29])
	↑ in PPAR*α*−/− mice [48]	
	↓ [50]	
	↑ [41, 49]	
	Human data: ↑ ([46, 47, 51]; reviewed in [29])	

LG100268	No change [44]	↓ [36, 40, 53, 54] Note that [36] shows thyroid hormone axis suppression with oral administration but not with intracerebroventricular administration. References [53, 54] investigate the mechanism of suppression of thyroid hormone axis by rexinoids
	↓ [30, 32, 34, 50]	
	↑ [36, 40, 41]	
	Note that [36] shows increased TG with oral administration but not with intracerebroventricular administration	

AGN194204	↑ [39, 42]	↓ [42]

LG101506	No change in Sprague Dawley rats; ↑ at low doses and no change at higher doses in Zucker rats, with effects most evident on day 7 of a 14-day treatment period [40]	No change in either Sprague Dawley or Zucker rats [40]
